# A CDC42-centered signaling unit is a dominant positive regulator of endothelial integrity

**DOI:** 10.1038/s41598-017-10392-0

**Published:** 2017-08-31

**Authors:** J. Amado-Azevedo, N. R. Reinhard, J. van Bezu, R. X. de Menezes, V. W. van Beusechem, G. P. van Nieuw Amerongen, V. W. M. van Hinsbergh, P. L. Hordijk

**Affiliations:** 1 Department of Physiology, Institute for Cardiovascular Research, VU University Medical Center, Amsterdam, The Netherlands; 2 Department of Molecular Cytology, Swammerdam Institute for Life Sciences, van Leeuwenhoek Centre for Advanced Microscopy, University of Amsterdam, Amsterdam, The Netherlands; 3 Department of Epidemiology and Biostatistics, VU University Medical Center, Amsterdam, The Netherlands; 4 Department of Medical Oncology, RNA Interference Functional Oncogenomics Laboratory, VU University Medical Center, Amsterdam, The Netherlands

## Abstract

Endothelial barrier function is carefully controlled to protect tissues from edema and damage inflicted by extravasated leukocytes. RhoGTPases, in conjunction with myriad regulatory proteins, exert both positive and negative effects on the endothelial barrier integrity. Precise knowledge about the relevant mechanisms is currently fragmented and we therefore performed a comprehensive analysis of endothelial barrier regulation by RhoGTPases and their regulators. Combining RNAi with electrical impedance measurements we quantified the relevance of 270 Rho-associated genes for endothelial barrier function. Statistical analysis identified 10 targets of which six promoted- and four reduced endothelial barrier function upon downregulation. We analyzed in more detail two of these which were not previously identified as regulators of endothelial integrity. We found that the Rac1-GEF (Guanine nucleotide Exchange Factor) TIAM2 is a positive regulator and the Cdc42(Rac1)-GAP (GTPase-Activating Protein) SYDE1 is a negative regulator of the endothelial barrier function. Finally, we found that the GAP SYDE1 is part of a Cdc42-centered signaling unit, also comprising the Cdc42-GEF FARP1 and the Cdc42 effector PAK7 which controls the integrity of the endothelial barrier. In conclusion, using a siRNA-based screen, we identified new regulators of barrier function and found that Cdc42 is a dominant positive regulator of endothelial integrity.

## Introduction

The integrity of blood vessels is critical to normal physiology and organ function as it is pivotal for continued circulation and proper distribution and delivery of nutrients and oxygen to the tissues. Control of the exchange of fluid, macromolecules and leukocytes occurs via the sealed monolayer of endothelial cells (ECs) and its surface glycocalyx^1^, primarily by vesicular transport across the ECs or via specific leukocyte receptors, respectively. Furthermore, endothelial permeability is regulated by vasoactive agents, inflammatory mediators and growth factors as well as by biomechanical stimuli. These induce opening of the endothelial junctions, thus facilitating leakage of plasma proteins^2, 3^. Hyperpermeability can become life-threatening in sepsis, shock and adult respiratory distress syndrome. Consequently, preservation of endothelial integrity is controlled at multiple levels, in particular at endothelial junctions and the F-actin cytoskeleton^4^. This occurs by vessel stabilizing agonists and by intracellular signaling pathways that either enforce barrier properties or balance intracellular events triggered by vessel-disrupting stimuli. Better understanding of these regulatory pathways can help to improve endothelial stabilization, when needed. In most continuous endothelia in the body, except the brain^5^, the endothelial monolayer is sealed by a belt of adherens junctions, which contain VE-cadherin homodimers as the main cell-cell contact molecule^6, 7^. In the adherens junction complex, VE-cadherin is connected to the F-actin cytoskeleton through catenins. Changes in cytoskeletal dynamics which may increase contractile forces are causally linked to the loss of VE-cadherin barrier function^8, 9^ and increased vascular leakage^10^.

The molecular mechanisms that govern ECs contraction are coordinated by small GTP-binding proteins of the Rho family. This group of approximately 20 proteins are differentially expressed and, despite relatively high sequence homology, exert different effects on cytoskeletal dynamics, feeding into cell polarity, adhesion, migration and, in EC﻿s﻿, barrier function^11, 12^. RhoGTPases act as switches that are ‘off’ when bound to GDP and ‘on’ when bound to GTP. The ‘on’ state allows interaction with effector proteins and the initiation of downstream signaling. The transition between these states has been intensely studied over the past 25–30 years which uncovered a surprising number of RhoGTPase-regulating molecules that serve to bring Rho proteins in either the ‘on’ or ‘off’ state^13, 14^. They comprise Guanine nucleotide Exchange Factors (RhoGEFs) which promote the exchange of GDP for GTP, switching the RhoGTPase to ‘on’; and GTPase Activating Proteins (RhoGAPs), which stimulate the intrinsic hydrolysis of GTP, which results in a GDP-bound (‘off’) RhoGTPase. Furthermore, Guanine nucleotide Dissociation Inhibitors (RhoGDIs) are cytosolic chaperones that maintain many of the RhoGTPases in their ‘off’ state, also protecting them from proteasomal degradation^15^. There exist ~80 RhoGEFs, ~65 RhoGAPs and 3 RhoGDI’s^16, 17^. In addition, there is a large group of effector proteins, some of which interact with several related homologous Rho proteins once they are in the GTP-bound state.

The regulation of endothelial barrier function by RhoGTPases Rac1, RhoA and Cdc42 has been studied extensively^18–23^. In short, the Rac1 GTPase activates, under most circumstances, intracellular signaling which stabilizes the barrier. This is mostly due to the induction of membrane protrusion, which promotes cell-cell contact, together with counteracting the induction of contractility. In contrast, RhoA is best known for its stimulation of acto-myosin mediated contractility of F-actin stress fibers, which leads to reduced cell-cell contact, formation of intercellular gaps and increased permeability. However, RhoA is also important for the maintenance of inter-endothelial junctions (IEJ)^24, 25^ and was more recently associated with the closure rather than opening of small gaps at junctional protrusions^26^. Moreover, RhoA counteracts Rac1 signaling while Cdc42 signals in an analogous way as Rac1, and promotes the formation of small membrane protrusions, known as filopodia^27^.

Regarding Rho-regulating proteins, the situation is more complex, if only because of their large numbers^23^. Several GEFs and GAPs have been implicated in pathways disrupting or stabilizing EC integrity, in general due to their activation of Rac1 or RhoA. However, the relative importance in EC integrity of most of these regulatory proteins is unknown. To address this, we performed a siRNA-screen (270 targets) in primary human EC, based on the loss of expression of 82 RhoGEFs, 66 RhoGAPs, 3 RhoGDIs and 97 effector- and Rho-associated proteins. We also included 22 RhoGTPase family members. The primary readout of the screen was gain or loss of basal endothelial barrier function, as quantified using Electric Cell-substrate Impedance Sensing (ECIS). We subsequently performed a more in-depth analysis of a selection of the top-hits from the screen that promote or reduce endothelial barrier function. Our combined analysis revealed that a Cdc42-centered signaling unit, comprising the Cdc42GEF FARP1, the Cdc42GAP SYDE1 and Cdc42 effector proteins, including PAK7, is the most dominant, positive regulator of endothelial integrity.

## Results

### RhoGTPases and the functional siRNA screen

**Figure 1:**
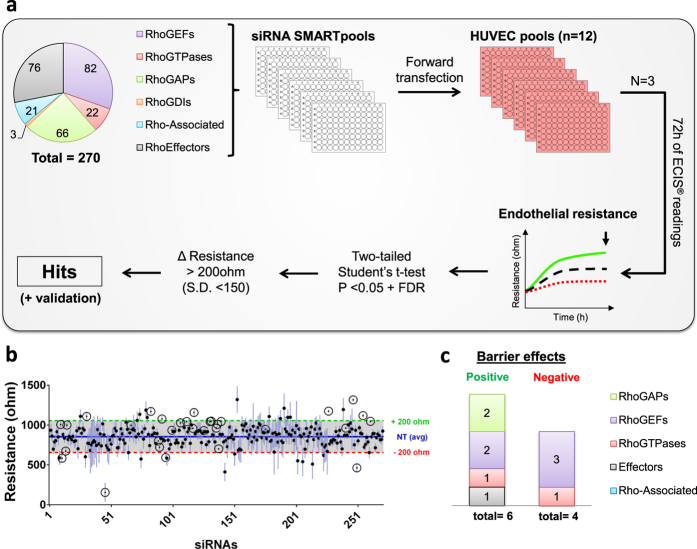
Functional RhoGTPases siRNA screen identifies new regulators of the endothelial barrier. (**a**) Schematic representation of the siRNA screen. Upper left corner, clockwise: the selected 270 siRNA targets distributed by functional group: RhoGTPases (22), RhoGEFs (82), RhoGAPs (66), RhoGDIs (3), Rho-effector proteins (76) and Rho-associated genes (21). 270 SMARTpools and control siRNAs were forward transfected into primary HUVECs seeded on 96W10idf ECIS arrays (n = 3 independent experiments). Endothelial barrier resistance was measured for 72 h following transfection by ECIS. Typical graph of the ECIS readout is shown. Depicted in green are exemplary readings of genes that enhance barrier function after knock-down; in red, genes that decrease barrier function upon loss-of-function; in black, a control non-targeting siRNA. Statistical analysis was performed and hits were identified as detailed in Results. Validation experiments were performed on the top hits. (**b**) Overview of individual mean endothelial resistance values registered at 72 h post-transfection of each siRNA pool (black dots) (n = 3). The average resistance of the non-targeting siRNA controls is represented by the background blue line. (n = 54). In grey, the threshold value set at ±200 ohm on the average non-targeting siRNA controls. Statistically significant hits, not all of which also induced large deviations in barrier function, are indicated by circles. (**c**) Distribution of 10 top hits by functional group.

To identify novel regulators of the endothelial barrier, we screened a custom library of siRNA SMARTpools (Dharmacon) targeting Rho-associated genes in freshly isolated HUVECs (human umbilical vein endothelial cells). The selection of siRNA targets was based on published data, resulting in a total of 270 target genes included in the library (Table S1). These targets were members of the 6 main classes of RhoGTPases and their regulatory proteins: 82 RhoGEFs, 22 RhoGTPases, 66 RhoGAPs, 3 RhoGDIs, 21 Rho-associated proteins and 76 effector proteins (Fig. 1a). The Electrical Cell-substrate Impedance Sensing (ECIS) system was used to measure endothelial barrier function of primary HUVECs, transfected with the individual siRNA pools. Three independent screens, including all 270 targets, were performed with three different pools of HUVECs, each derived from 12 different donors. The electrical endothelial impedance (resistance) of the monolayers was measured for 72 hours post-transfection and barrier function was evaluated (see schematic overview on Fig. 1a and Methods section). For data analysis, the mean endothelial resistance induced by each target siRNA in the three screens was compared to the mean endothelial resistance of non-targeting (NT) siRNA controls used in all plates (n = 54). Statistical tests were used to identify candidate hits: Student’s T-test with P < 0.05 followed by multiple test correction with False Discovery Rate (FDR) set to <0.05. This two-step analysis revealed 31 candidate hits (Fig. 1b) of which 10 are high confidence hits. To find relevant hits for the regulation of the endothelial barrier, we further filtered the statistical data by applying a threshold of ≥200 Ω to the mean resistance of the siRNA targets and a limit of 150 Ω of standard deviation values. This resulted in a list of 10 candidate genes that included novel and known regulators of the endothelial barrier (Fig. 1c and Table I). Six siRNAs promoted enhancement of the endothelial barrier resistance, namely: ArhGAP45 (HMHA1) and SYDE1 (RhoGAPs), DEF6 and PLXNB2 (RhoGEFs), mDia (effector protein) and RhoB (RhoGTPase). In contrast, four siRNAs decreased the endothelial barrier: TIAM2, TEM4 and FARP1 (RhoGEFs) and CDC42 (RhoGTPase) (Supplemental Fig. S1b,c).

For validation purposes, new siRNA SMARTpools of the ten hits were analyzed another two times in the ECIS assay following the exact same protocol but using different pools of HUVECs. In total, all 10 hits were confirmed by displaying consistently the same endothelial barrier response. Knock-down efficiency of all these hits was determined by qPCR and/or Western blot. Based on these results and because of their novelty in this field, we further studied two targets that showed opposite effects on the barrier function: T-Cell Lymphoma Invasion And Metastasis 2 (TIAM2 - RhoGEF) and Synapse Defective1 (SYDE1 - RhoGAP). In-depth analysis of the ArhGAP45 (HMHA1) protein and its role in endothelial barrier function will be part of a separate manuscript.

### TIAM2 is essential for endothelial barrier and cell-cell contacts maintenance

**Figure 2:**
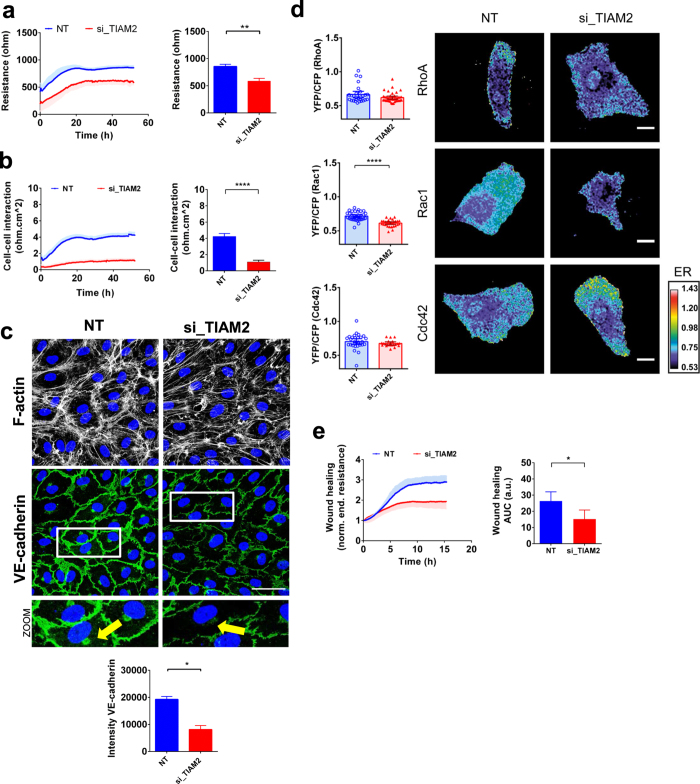
The RhoGEF TIAM2 is a positive regulator of the endothelial barrier. (**a**) Absolute endothelial electrical resistance of monolayers of HUVECs transfected with si_TIAM2 and non-targeting control siRNA, 16 h after transfection medium was refreshed; Bar graph: quantification of the 72 h post-transfection time-point. **P < 0.01 in two-tailed Student’s t-test (n = 3). (**b**) Absolute endothelial electrical resistance attributable to cell-cell interaction (Rb) of monolayers of HUVECs transfected with si_TIAM2. Quantification of the 72 h post-transfection time-point. ****P < 0.0001 in two-tailed Student’s t-test (n = 3 ± SEM). (**c**) Immunofluorescence staining of F-actin (white) and VE-cadherin (green) of HUVECs transfected with si_TIAM2 and non-targeting control siRNA for visualization of F-actin fibers and cell-cell contacts. Scale bar represents 50 μm. Representative images of n = 3–4 experiments. Arrow points to the difference of VE-cadherin intensity at the perinuclear region. Bar graph: Quantification of total VE-cadherin fluorescence per field of view. *P < 0.05; two-tailed Student’s T test. (**d**) YFP/CFP ratios of HUVECs transfected with si_TIAM2 and non-targeting control siRNA (n = 30) and either the RhoA- (n = 40), Rac1- (n = 32) or Cdc42- (n = 20) FRET sensor. Bar graphs depict mean FRET ratios in transfected HUVECs and error bars the mean 95%CI. Images show ratiometric images of representative cells. Warm colors represent high YFP/CFP ratios (Emission ratio – ER) ****P < 0.0001; two-tailed Student’s T-test. (**e**) Absolute endothelial electrical resistance of HUVECs transfected with si_TIAM2 after electrical wound; Lateral: Area under the curve for quantification of the total wound healing response. (n = 3) *P < 0.05 in in two-tailed Student’s T test.

HUVECs transfected with siRNA targeting TIAM2 displayed, already during the 16 hours of transfection reaction, a weakened endothelial barrier (25% reduction) as compared to the NT siRNA controls. This effect was stable and persisted throughout the entire measurement, i.e. up to 72 hrs (Fig. 2a). A similar response was observed with human MVECs (human microvascular endothelial cells) and HAECs (human aortic endothelial cells), albeit to a lesser extent (Supplemental Fig. S2b,c). Knock-down efficiency in HUVECs was determined by qPCR and showed >70% decrease in TIAM2 mRNA expression (Supplemental Fig. S2a). Modeling the ECIS resistance data into the Rb and Alpha parameters showed that silencing TIAM2 strongly decreased junctional resistance (i.e. cell-cell contacts; Rb, Fig. 2b) but did not interfere with cell-matrix interaction (Alpha, Supplemental Fig. S2d). Immunostaining of the cell-cell junction protein VE-cadherin, a key regulator of endothelial barrier function, revealed that knock-down of TIAM2 not only significantly decreased the intensity of VE-cadherin staining but also altered its distribution at the junctional membrane as observed by the non-linear, jagged, staining pattern (Fig. 2c). This decrease was also observed at the protein level (Supplemental Fig. S2e – full length blots are presented in the supplementary information section). The intensity of VE-cadherin staining in the perinuclear region was strongly decreased in TIAM2 depleted cells (Fig. 2c zoomed images, yellow arrows) The F-actin staining intensity was reduced in si_TIAM2 transfected cells but quantification showed that this effect did not reach significance. Surprisingly, silencing the close homolog TIAM1, which was previously implicated in the Rac1-mediated regulation of endothelial integrity showed no effect on the basal endothelial barrier (Supplemental Fig. S2f).

### TIAM2 regulates Rac1 activity in resting endothelial cells

TIAM2 is a known RhoGEF (Guanine nucleotide Exchange Factor)^28, 29^ and its silencing impaired the endothelial barrier function. To understand the contribution of the main RhoGTPases (RhoA, Rac1 and Cdc42) to this endothelial phenotype, we measured their activation by using FRET-based single-chain biosensors in live TIAM2 knock-down cells^30–34^. These FRET sensors monitor RhoGTPase activation where an increase in YFP/CFP ratio corresponds to an increased activation. TIAM2 knockdown cells, expression the Rac1 FRET sensor showed significantly lower YFP/CFP ratios compared to NT siRNA control cells (Fig. 2d, middle panel). In contrast, Cdc42 and RhoA FRET sensor expressing cells showed no significant differences in YFP/CFP ratio, suggesting that TIAM2 does not activate Cdc42 or RhoA (Fig. 2d, upper and lower panels). These results indicate that in resting HUVECs, the RhoGEF TIAM2 acts as an exchange factor for Rac1 and not for RhoA or Ccd42, which is in line with previous data^35^. The activity levels of RhoA were further confirmed by the biochemical G-LISA assay, where a small but not statistically significant increase in activity was found (Supplemental Fig. S2h). This suggests that the reduction in Rac1 activation following the loss of TIAM2 allows limited activation of RhoA by a cognate RhoAGEF. Taken together, these data indicate that in the resting endothelium, the GEF TIAM2 is a constitutive, positive regulator of the barrier function through its activation of Rac1.

### Silencing TIAM2 reduces endothelial wound healing ability

In addition to analyzing the effect of silencing TIAM2 expression on basal monolayer resistance, we investigated whether loss of TIAM2 also influenced wound healing, as assessed using the ECIS wounding protocol. TIAM2-depleted cells showed impaired ability to heal the electrical wound and their rate of migration to heal the wounded area was correspondingly slower (Fig. 2e). Quantification of the wound healing response (area under the curve) showed a significant reduction in HUVECs transfected with the siRNA targeting TIAM2 as compared to cells transfected with the control NT siRNA. These findings further support the role of TIAM2 as a constitutive Rac1 activator in endothelial cells.

### SYDE1 is a negative regulator of the endothelial barrier

**Figure 3:**
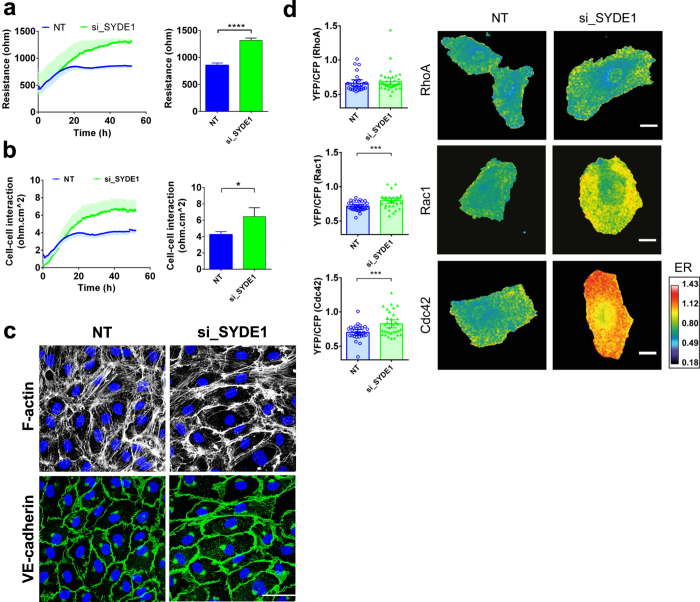
The RhoGAP SYDE1 is a novel negative regulator of the endothelial barrier. (**a**) Absolute endothelial electrical resistance of HUVEC monolayers transfected with si_SYDE1 and non-targeting control siRNA, 16 h after transfection medium was refreshed. Bar graph: endothelial resistance quantification at 72 h post-transfection ***P < 0.001 in two-tailed Student’s t-test (n = 3); (**b**) Absolute endothelial electrical resistance attributable to cell-cell interaction (Rb) of monolayers of HUVECs transfected with si_SYDE1 and non-targeting siRNA control. Respective endothelial resistance quantification at 72 h post-transfection. *P < 0.05 in two-tailed Student’s t-test (n = 3 ± SEM). (**c**) Immunofluorescence staining of F-actin (white) and VE-cadherin (green) for visualization of F-actin fibers and cell-cell contacts of HUVECs transfected with si_SYDE1 and non-targeting siRNA control. Scale bar represents 50 μm. Representative images of 3–4 experiments. (**d**) YFP/CFP ratios of HUVECs transfected with si_SYDE1 and non-targeting control siRNA (n = 30) and either the RhoA- (n = 40), Rac1- (n = 29) and Cdc42- (n = 34) FRET sensor. Bar graphs depict mean FRET ratios in transfected HUVEC and error bars the mean 95%CI. Images show ratiometric images of representative cells. Warm colors represent high YFP/CFP ratios (Emission ratio – ER) ***P < 0.001; two-tailed Student’s T-test.

The second hit from the screen which we investigated further was SYDE1. Among all the hits identified in this screen, SYDE1 showed the strongest effects on endothelial barrier function. HUVECs transfected with siRNA targeting SYDE1 showed a significant increase in endothelial barrier resistance (42% increase) when compared to NT siRNA controls (Fig. 3a). Knock-down efficiency was determined by qPCR and SYDE1 mRNA expression levels detected after transfection, were reduced by 55% (Supplemental Fig. S3a). Similarly, silencing of SYDE1 in human MVEC and HAECs increased the endothelial electrical resistance by 25% and 15%, respectively (Supplemental Fig. S3b–c). Upon modeling of the ECIS resistance readings, data showed that silencing SYDE1 significantly enhanced the junctional resistance (Fig. 3b) and only slightly increased cell-matrix interactions (Supplemental Fig. S3d). Immunostaining of VE-cadherin revealed that knock-down of SYDE1 did not induce major changes in cell morphology or junction stability as observed by the thin reticular distribution of VE-cadherin (Fig. 3c and Supplemental Fig. S3e–g; full length blot is presented in in supplementary information section). The F-actin intensity did not change albeit that we detected a relative accumulation of F-actin fibers in the cortical area of the cells.

### SYDE1 is a GAP for Cdc42 and Rac1 on endothelial cells

To understand the mechanism behind the effect of SYDE1 on barrier function, we evaluated which of the major RhoGTPases RhoA, Rac1 or Cdc42 were affected after silencing SYDE1. To that end, we used the single-chain FRET biosensors in HUVECs and compared SYDE1 depleted- and NT siRNA control cells. Both Rac1- and Cdc42-expression FRET sensor cells showed a significant increase in YFP/CFP ratio upon knock-down of SYDE1 whereas no differences in YFP/CFP were observed in the RhoA FRET sensor expressing cells (Fig. 3d). In addition, we used the G-LISA assay for RhoA to validate the FRET data and found accordingly that there were no significant differences between controls and cells lacking SYDE1 (Supplemental Fig. S3h). These results indicate that SYDE1 is a GAP for both Rac1 and Cdc42, but nor for RhoA which acts as a negative regulator of the endothelial barrier function.

### A CDC42-centered signaling unit is a key regulator of basal endothelial barrier function

**Figure 4:**
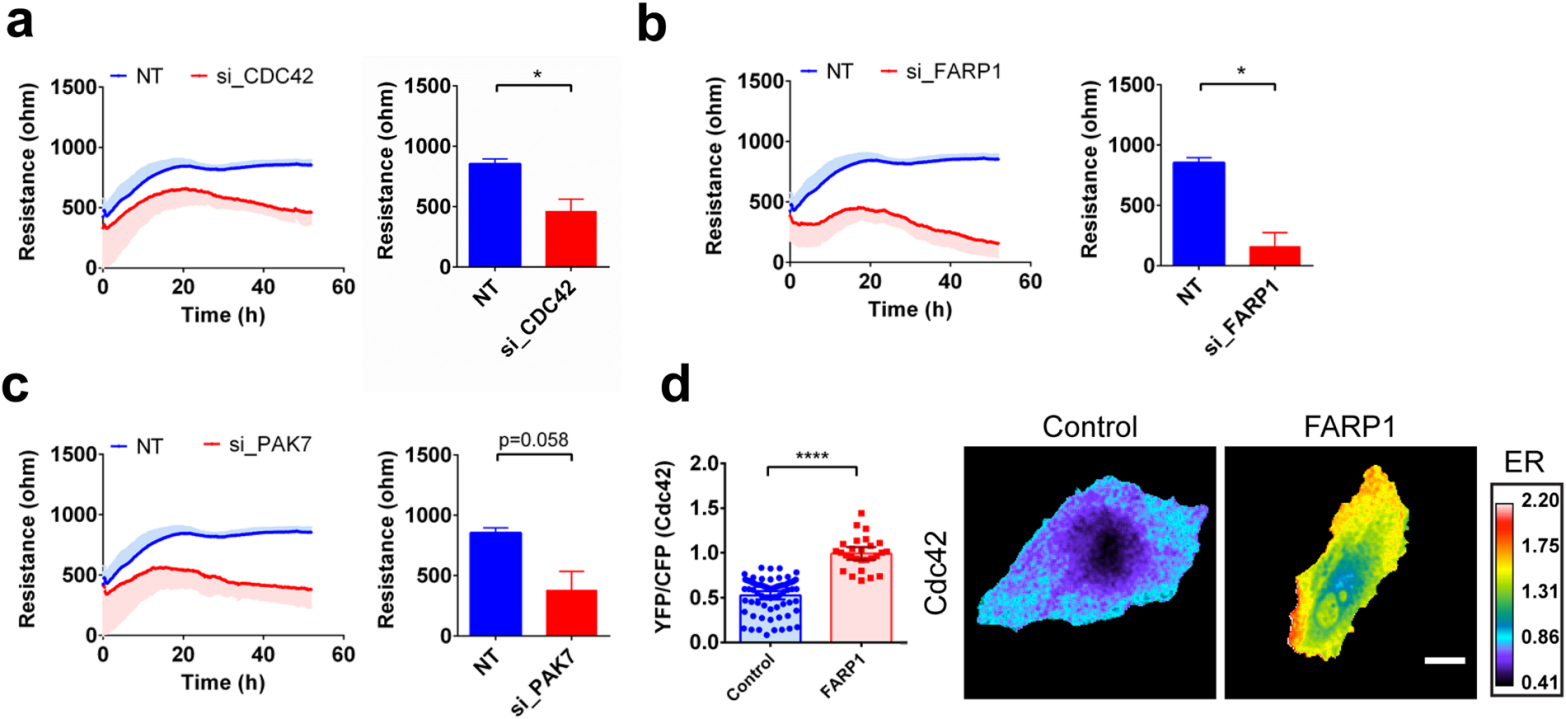
CDC42 related genes essential for endothelial barrier maintenance. Absolute endothelial electrical resistance of HUVECs transfected with siRNA targeting (**a**) the RhoGTPase CDC42, (**b**) the Cdc42GEF FARP1 and (**c**) the effector proteins PAK7 16 h after transfection medium was refreshed. (n = 3 for all experiments); Bar graphs: quantification at 72 h post-transfection. *P < 0.05 in two tailed Student’s T test. (**d**) YFP/CFP ratios of Cdc42 FRET sensor-expressing HUVECs co-expressing either mCherry control (n = 70) or mCherry FARP1 (n = 27). Error bars depict 95%CI. On the right, representative ratiometric image. Warm colors represent high YFP/CFP ratios (Emission ratio – ER) ****P < 0.0001 in two-tailed Student’s T-test. Scale bar 20 μm.

During the analysis of the results of our screen, we noticed that several of the most significant hits showed a functional connection to the RhoGTPase Cdc42^29, 36–40^. Subsequently, we therefore focused on Cdc42 regulatory proteins. Not only the Cdc42-GAP SYDE1 and Cdc42 itself were among the significant hits identified but also the Cdc42-GEF FARP1 (FERM, ARH/RhoGEF And Pleckstrin Domain Protein 1) (Table 1). Silencing Cdc42 as well as FARP1 induced a strong, negative effect on endothelial barrier function (Fig. 4a,b) as well as on cell morphology. Expression of mCherry-FARP1 in Cdc42 FRET sensor expression cells showed a significant increase in YFP/CFP ratio compared to mCherry control cells (Fig. 4d). This confirms that FARP1 can act as a Cdc42GEF in ECs. Additional FRET sensor experiments showed that ectopic expression of mCherry-FARP1 also could activate RhoA, but not Rac1 (Supplemental Fig. S4a,b).

**Table 1:** Overview of the significant positive and negative regulators of the endothelial barrier identified in this functional siRNA screen.

siRNA target	Class	At 72 h post-transfection			
		Resistance mean ± SD (Ω) (n = 3)	Δ Resistance (Ω)	2 tailed t-test *P-value*	False discovery rate (FDR)
ArhGAP45*	GAP	1174.4 ± 25	319.3	1.9E-07	2.56E-05
SYDE1*	GAP	1316.4 ± 34	461.3	5.97E-06	0.000537
RHOB	RHO	1118.2 ± 32	263.2	7.69E-05	0.002964
DEF6*	GEF	1108.5 ± 51	253.5	0.005144	0.101512
PLXNB2*	GEF	1170.1 ± 59	315	0.005837	0.101512
TIAM2*	GEF	592.8 ± 51	−262.3	0.004621	0.101512
DIAPH1*	EFF	1157.1 ± 57	302	0.005582	0.101512
CDC42	RHO	462.9 ± 80	−392.1	0.011879	0.168803
TEM4	GEF	583.9 ± 70	−271.1	0.017703	0.207813
FARP1*	GEF	138.4 ± 144	−716.6	0.024394	0.260555

*Genes identified in this screen as novel regulators of the endothelial barrier.

Having identified FARP1/CDC42/SYDE1 as possible key regulators of endothelial integrity, we searched our list of hits for potential effector proteins of this barrier-regulating signaling unit. P21 activated kinases (PAKs) are Ser/Thr kinases that are well-described effector proteins for Rac1 and Cdc42 GTPases^41–43^. There are five isoforms of PAKs that were all included in our initial screen. siRNA-mediated loss of PAK1, PAK2, PAK4 and PAK6 showed only very subtle effects on the basal endothelial barrier. Interestingly, silencing PAK3 had positive effects on the endothelial barrier, by increasing its electrical resistance 19% (Supplemental Fig. S4c). However, PAK7 (a.k.a. PAK5) was the only isoform of which the downregulation caused an effect comparable to the loss of Cdc42 (Fig. 4c).

PAK7 was described to be a Cdc42 effector^40^ which is linked to control of regulation of actin dynamics^44, 45^, but which was not previously connected to endothelial barrier regulation. Silencing PAK7 expression in HUVECs induces a significant loss of barrier function (Fig. 4c). This, in line with the effects induced by downregulation of Cdc42 and FARP1 described above, suggests that PAK7 acts as a Cdc42 effector which is, like Cdc42 itself^46^, required for maintenance of actin-regulated endothelial integrity.

**Figure 5:**
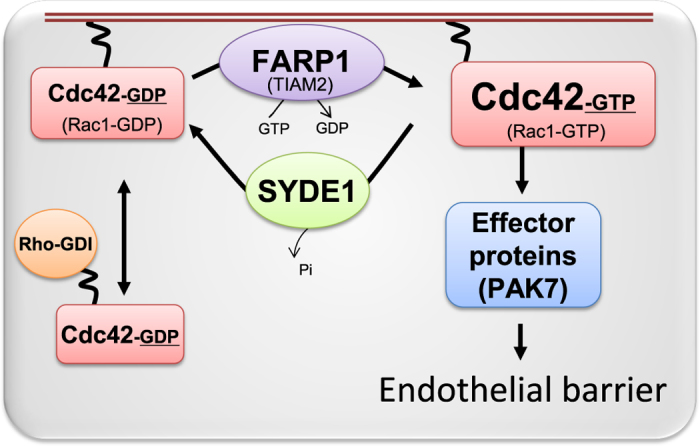
CDC42 is a main regulator of barrier function in resting endothelial cells. Proposed model of Cdc42 regulation of endothelial barrier function. The Cdc42-GEF FARP1 activates Cdc42, which subsequently activates effector proteins, including PAK7, which mediate stabilization of the endothelial barrier. The RhoGAP SYDE1, once activated, will inactivate Cdc42, thus reverting the positive regulation of the endothelial barrier. Moreover, the Rac-GEF TIAM2 activates Rac1 which leads to a stable endothelial barrier. However, like its homolog TIAM1, it might also activate Cdc42.

In summary, these findings led us to propose a model of regulation of endothelial barrier function in normal primary human ECs: the FARP1-GEF may activate Cdc42, which signals to its effectors, including PAK7, which in turn promote a stable and functional endothelial barrier. This signaling can be balanced by SYDE1 which acts as a Cdc42GAP, inactivating the GTPase and thereby allowing junctional dynamics. If this SYDE1-mediated inactivation becomes persistent, the negative consequences for the endothelial barrier and cell morphology are very disruptive as can be deduced from the loss of Cdc42 and FARP1 (Fig. 4a,b). This data suggests that Cdc42 and its associated regulatory and effector proteins play a crucial, non-redundant role in the regulation of endothelial barrier function. Based on these findings, we suggest a model in which all the above described regulatory cues are combined (Fig. 5). Currently, the model is based in part on current data and in part on correlations in functional responses. The precise contribution of the various components and level of redundancy under different conditions will require further investigation.

## Discussion

In this study, we identified new RhoGTPase-related regulators of the endothelial barrier by using a quantitative and highly reproducible siRNA-based screen with transendothelial electrical resistance measured by ECIS as a readout. Out of 270 RhoGTPase-associated genes screened, six regulate the endothelial barrier negatively (ArhGAP45, SYDE1, RHOB, DEF6, PLXNB2 and DIAPH1) and four positively (TIAM2, CDC42, TEM4 and FARP1). In addition to identifying TIAM2 as a novel regulator of endothelial integrity through its activation of Rac1, we found that Cdc42 and a number of its regulatory proteins (SYDE1, FARP1 and PAK7) showed the most prominent effects on the maintenance of the endothelial barrier.

This type of systematic screen was previously done either focusing on specific classes of proteins, such as GEFs, GAPs, Effectors and GTPases^47–52^ or on the genome^53, 54^. Considering such high numbers of regulatory proteins (c.a. 150)^23^ involved in RhoGTPase signaling cascades, we expected to detect significant redundancy between proteins. Indeed, silencing most of these regulatory proteins did not affect the basal endothelial barrier. However, knock-down of several genes did affect the endothelial barrier and interestingly did so in opposite ways. The class distribution of the hits by type of response showed that both GAPs (ArhGAP45 and SYDE1) and GEFs (DEF6 and PLXNB2) negatively regulate the endothelial barrier. The same applies to the only RhoGTPase, the loss of which induced a barrier-enhancing effect, i.e. RhoB, a known negative controller of Rac1 in barrier restoration after thrombin-induced loss of integrity^55^. Moreover, we also confirmed the effector protein DIAPH1, (diaphenous1), one of the main effector proteins of RhoA, as a known regulator of the endothelial barrier^24^. Unexpectedly, loss of well-established regulators like RhoA or Rac1 did not induce significant changes in basal monolayer resistance. We reasoned that for RhoA to play a significant and detectable role in the regulation of the endothelial barrier, an activation of the endothelium had to occur, for example by vaso-mediators such as thrombin, histamine or others^56^. This would also explain why known exchange factors like GEF-H1^57^, LARG^58^, VAV2^59^ or TRIO^60^ appear not to play a critical role in the maintenance of basal endothelial barrier function. Regarding Rac1 we hypothesize that either a small percentage of protein that is left upon siRNA transfection is sufficient to sustain the EC barrier or that the loss of Rac1 is compensated by an increase (in activity) of perhaps RhoG, considering their overlapping pathways^61^, or Cdc42, thus masking the function of Rac1 in endothelial barrier function. We also expected an effect of the loss of TIAM1, which is a Rac1 exchange factor and has been reported to improve endothelial barrier function^62^. Unexpectedly, however, we identified its homologue TIAM2 as a barrier regulating GEF.

TIAM2 (also known as STEF) is known to have a variable expression pattern in HUVECs^16^ but our data show that it was expressed in our set of primary cells and that it is relevant for EC function. HUVECs that lack TIAM2 show, besides a weaker barrier function, a decrease in VE-cadherin accumulation at the plasma membrane. These observations were confirmed by very low Rb (cell-cell interaction) values detected by the ECIS upon downregulation of TIAM2. This effect was also observed in microvascular and arterial endothelial cells but to a lower extent. Whether this is due to lower expression levels of TIAM2 in these types of cell or to compensatory effects of its homolog TIAM1 remains to be established. Zhao and colleagues^63^ showed that TIAM2 silencing impaired migration and invasion ability in non-small cell lung cancer cells. Our findings also demonstrate that cells lacking TIAM2 could not efficiently heal the wound in the ECIS wounding assay. This highly reproducible method^64–67^ has been established before and provides quantitative data similar to other methods^64^. TIAM2 has been shown by Chiu *et al*.^29^ to be a Rac1 exchange factor, a notion which is supported by our FRET-biosensor analysis. The fact that, in contrast to TIAM2, loss of Rac1 had little effect on endothelial barrier function was unexpected, knowing its important role on the establishment of the adherens junctions^68^. Possibly, TIAM2 activates other RhoGTPases, in particular Cdc42, as previously suggested for TIAM1^69^ or for RhoG as shown for VAV2, VAV3 and TRIO^60, 70, 71^. However, our data do not support activation of Cdc42 by TIAM2 (compare Fig. 2d), suggesting that TIAM2 acts through Rac1 in a non-redundant manner.

Interestingly, the two top hits from our screen are RhoGAPs of which not much is known. It was only recently found that ArhGAP45 (a.k.a. HMHA1) encodes a functional RhoGAP domain^72^ and that it is expressed in HUVECs^16^. The effects of its loss will be described in more detail in a separate manuscript. Loss of SYDE1, which is also expressed in HUVECs^16^ promoted the highest barrier resistance levels among all 270 targets analyzed. However, silencing SYDE1 by siRNA was only partially efficient as values of mRNA expression were decreased by 55% only. Nonetheless, even with an incomplete knock-down, the effects on the endothelial barrier phenotype are striking.

Recently, SYDE1 was shown to be highly expressed in human placentas and to be directly related to cytoskeletal remodeling and cell invasion and migration^36^. In line with this, our findings show that endothelial cell-cell contacts are enhanced upon silencing SYDE1 expression. SYDE1 silencing in human ovarian carcinoma cell line has been shown to increase E-cadherin^73^. However, we found no changes in VE-cadherin expression using either immunostaining or analysis of protein expression. We also detected increased wound healing ability in the absence of SYDE1, albeit that statistical significance was not reached (Supplemental Fig. S3g). Finally, use of FRET-biosensors showed that loss of SYDE1 in HUVECs led to increased activity of Cdc42, followed by Rac1. This data support that, in HUVECs, SYDE1 is preferentially a GAP for Cdc42 but can also inactivate Rac1.

We noticed that among our hits there were additional CDC42-related genes, namely the GEF FARP1 and Cdc42 itself. FARP1 is expressed in different types of ECs, both microvascular^74^ and HUVECs^16^. Loss of SYDE1 or FARP1 results in completely opposite effects on the endothelial barrier. As mentioned above, SYDE1 knock-down enhanced-, whereas FARP1, like Cdc42, decreased endothelial barrier resistance. Moreover, ectopic expression of FARP1 confirmed the GEF activity towards Cdc42 in HUVECs; we did not detect activation of Rac1. However, there is conflicting data regarding FARP1 substrate specificity. It was previously suggested to be a Rac1-GEF^75^ or even a RhoA-GEF^76^ in other cell types. In our FRET-assays, FARP1 expression also activated RhoA (Supplemental Fig. S4a), but since its downregulation mimics the loss of Cdc42, but not of RhoA, FARP1 may act as a Cdc42GEF in the control of barrier function, in line with previous suggestions based on structural determinants^77^.

In addition to these two Cdc42 regulatory proteins (the Cdc42-GAP SYDE1 and the Cdc42-GEF FARP1) we focused on potentially relevant effector proteins. Detailed analysis of the individual endothelial resistance curves obtained with the ECIS, revealed that one (PAK7) fully resembled the Cdc42 and FARP1 effects. PAK family members are known effectors of Rac/Cdc42 GTPases. PAK7, also known as PAK5 has been reported to interact mainly with Cdc42 but also with RhoD and RhoH^40^. Out of the five different PAK-isoforms analyzed in our screen, only loss of PAK7 decreased the basal endothelial barrier resistance. Although we cannot exclude additional Cdc42 effector proteins to be relevant for endothelial integrity, under the conditions of this screen, PAK7 appears most closely functionally related to the other Cdc42 interacting proteins we identified.

In summary, we identified a series of novel regulators of endothelial integrity, which all are directly or indirectly connected to RhoGTPases. The screen was performed on monolayers of primary human ECs, cultured under standard conditions and not stimulated with cytokines or vaso-mediators. Our data are consistent with a model describing a signaling unit, centered around Cdc42, which preserves endothelial integrity under resting conditions (Fig. 5). In this model, the unit comprises, next to Cdc42, the GEF FARP1 and the GAP SYDE1 and the Cdc42 effector PAK7. The stabilization of endothelial integrity is further supported by the Rac1GEF TIAM2. Cdc42-regulated barrier function is counteracted by a barrier-destabilizing pathway, which presumably comprises one of the RhoA-related GTPases, possibly RhoB. Finally, preliminary data indicate that while Cdc42 is important for barrier function under resting conditions, this may well be different in activated ECs, for example during barrier restoration. Moreover, the components of the unit we propose in the model in Fig. 5, are clearly not the only ones which can perform GAP/GEF/effector functions towards Cdc42. Cell type, experimental conditions, location in tissues or along blood vessels etc., is likely to not only change relative expression of the various components, but also of related ones, thus balancing redundancy vs unique functionality. Future research is warranted to define the interplay between barrier stabilizing and -disrupting GTPases, their cognate regulators and effectors.

## Material and Methods

### Reagents and antibodies

The following antibodies were used for immunostaining: anti-VE-cadherin XP (Cell Signaling Technologies, Danvers, MA); Rhodamin-phalloidin for direct F-actin staining (Invitrogen Corporation, San Diego, CA) and DAPI (Thermo Fisher Scientific). Alexa 488 and Alexa 555 were the secondary antibodies (Thermo Fisher Scientific, Waltham, MA). For protein analysis: anti-VE-cadherin (Cell Signaling Technologies, Danvers, MA) and anti-β-Tubulin (Cell Signaling Technologies, Danvers, MA). Secondary antibodies were from Invitrogen, Paisly, UK.

### Endothelial cell culture

Human umbilical vein endothelial cells were freshly isolated from umbilical cords of healthy donors, as previously described^78^ and were obtained at the Amstelland Ziekenhuis (Amstelveen, The Netherlands). Written informed consent was obtained from all donors in accordance with the institutional guidelines and the Declaration of Helsinki. After isolation, cells of different donors were pooled and resuspended in M199 medium supplemented with 100 U/mL penicillin and 100 μg/mL streptomycin, 2 mmol/L L-glutamine (all Lonza, Belgium), 10% heat-inactivated human serum (Invitrogen, WI, USA), 10% heat-inactivated new-born calf serum (Lonza, Belgium), 150 μg/mL crude endothelial cell growth factor (prepared from bovine brains), 5 U/mL heparin (Leo Pharmaceutical Products, Breda, The Netherlands) and seeded on 1% gelatin coated plates. Cells were cultured at 37°C and 5%CO_2_ with change of medium every other day. For all experiments pools of HUVECs of at least 3 donors in passage 2 were used. All relevant protocols were reviewed and approved according to standard procedures by the Ethical Committee of the VU University Medical Center.

#### For FRET measurements

Primary HUVEC were obtained from Lonza and cultured on fibronectin-coated dishes in EGM-2 medium, supplemented with singlequots (Lonza, Verviers, Belgium). HUVEC at passage #4 were transfected with 25 nM of ON-TARGETplus (OTP) SMARTpool siRNAs Non-targeting Control Pool (#D-001810-10-05), SYDE1 (#L-015856-01-0005) and TIAM2 (#L-008434-00-0005) and 0.25% (v/v) Dhamarfect1 transfection reagent (#T-2001-03) (all Dharmacon/GE Heathcare, Lafayette, CO). In turn, HUVECs were transiently transfected with 2 μg plasmid DNA using an electroporation system (Invitrogen, MPK5000) and a corresponding microporation kit (Invitrogen). A single pulse was generated at 1300 V for 30 ms. After transfection, HUVECs were seeded on fibronectin-coated glass coverslips and grown to a monolayer.

### siRNA library and functional screen

A custom ON-TARGETplus SMARTpool library (Dharmacon/GE Healthcare, Lafayette, CO) targeting 270 human RhoGTPases and Rho-associated genes was ordered on 96-well microtiter plates (Table S.I). To minimize off-targets effects and maximize loss of gene function, pools of siRNA were chosen over individual sequences. siRNA pools consisted of four different siRNA duplexes against each target gene^79, 80^. siRNAs against ROCK1 (#L-003536-00-0005) and OTP Non-targeting Control Pool (#D-001810-10-05) were used as negative control whereas siRNA against ROCK2 (#L-004610-00-0005) as positive control (barrier decreasing effects) (Supplemental Fig. S1a) (all from Dharmacon/GE Healthcare, Lafayette, CO). Prior to the screens, optimization experiments were carried out and transfection efficiencies were calculated by measuring mRNA knockdown of control genes (>70%). We found in preliminary experiments that the transfection efficiency of the different controls was, on average, highest at 72 h post-transfection. We therefore selected this time-point for the screens and for the other assays in the study. Subconfluent passage 2 cells were seeded (1 × 10^4^ cells/well) on 1% gelatin-coated 96W10idf arrays (Applied Biophysics, Troy, NY) in complete M199 medium (as described above). Forward transfections were performed according to manufacturer’s instructions and using siRNAs at 25 nM final concentration and 0.25%(v/v) of Dharmafect 1 transfection reagent (Dharmacon/GE Healthcare, Lafayette, CO) in 100 μL total volume. After 16 h, medium was replaced by complete M199 medium (as described above). Confluence of the endothelial monolayers was preserved throughout the screens by means of seeding the same number of cells per well, checking the stability of the electrical signal of impedance and following the same standardized protocol. The screens (n = 3) were performed on different pools of HUVECs of 12 different donors. The siRNA screens were performed in collaboration with the RNA Interference Functional Oncogenomics Laboratory (RIFOL) at the VUmc Cancer Center Amsterdam.

### Endothelial barrier function measurements

Endothelial barrier function was measured by Electrical Cell-substrate Impedance Sensing (ECIS). Briefly, passage 2 cells (1 × 10^4^ cells/well) were seeded on 1% gelatin-coated 96w10idf or (1 × 10^5^ cells/well) 8w10E arrays connected to the ECIS®ZTheta Array Station (Applied Biophysics, Troy, NY) and transfected as described above. Throughout the first 90 h of the experiment (seeding, transfection and the following 72 h), endothelial resistance data was collected by multifrequency readings of: 1000 Hz, 2000 Hz, 4000 Hz, 8000 Hz, 16000 Hz, 32000 Hz and 64000 Hz. Barrier function assays were performed at 72 h post-transfection. Endothelial multifrequency resistance was modeled for the calculation of cell-cell adhesion (Rb) and to cell-matrix interaction (Alpha).

In this manuscript, the time-point zero hours in the endothelial barrier resistance graphics corresponds to the end of the 16 h transfection period, when transfection medium was replaced by complete M199 medium.

### Electric wound healing assay

Endothelial wound healing capacity was measured by using the ECIS. In short, passage 2 cells (1 × 10^5^ cells/well) were seeded on 1% gelatin-coated 8w10E arrays connected to the ECIS®ZTheta Array Station (Applied Biophysics, Troy, NY) and transfected as described above. At 72 h post-transfection, a pulse of high voltage and frequency was applied to the arrays (2 hits of 64000 V for 20 s) that led to cell-free gold electrodes (each electrode has a diameter of 250 μm). Subsequently, the rate of migration of neighboring cells to heal the wound was measured at multiple frequencies for the following 16 h.

### mRNA analysis

Transfected HUVECs were lysed and total RNA isolated using the RNeasy Microkit (Qiagen, Hilden, Germany) at 72 h post-transfection according to manufacturer’s instructions. Isolated RNA was reverse-transcribed to cDNA using the Cloned AMV First-strand cDNA synthesis kit (Invitrogen, San Diego, CA). cDNA was used for quantitative analysis of gene expression (qPCR) with Takyon low ROX SYBR Master Mix blue (Eurogentec, Seraing, Belgium). Primers used for qPCR analysis were designed using PubGene and PrimerBlast and HPRT1 (Hypoxanthine Phosphoribosyltransferase 1) was used as reference gene. Primers used: TIAM2 forward 5′-CTACCACCTGACGGAAGCAC-3′ and reverse 5′-TAGCTGGTCAAACACGGTCC-3′; SYDE1 forward 5′-TGTATGCCAAGCTGACCCTG-3′ and reverse 5′-TACAGTCCCACTACCCGCAG-3′; HPRT1 forward 5′-GACCAGTCAACAGGGGACAT-3′ and reverse 5′-AACACTTCGTGGGGTCCTTTTC-3′.

### Protein analysis

For protein analysis, cells were seeded in 5 cm^2^ culture wells and transfected as described above. At 72 h post-transfection cells were washed and incubated with 1% HSA-M199 for an hour. Additionally, cells were washed with cold PBS and whole-cell lysates were collected by scrapping the cells in protein lysis buffer containing phosphatase (PhosStop) and protease inhibitors (Complete) (all from Roche Applied Sciences, Basel, Switzerland). Protein samples were loaded on 8% or 12% SDS-PAGE gels, electrophoresed and transferred to nitrocellulose membranes. Membranes were blocked and protein analysis was performed by incubation with the above designated antibodies.

### G-LISA – RhoA activity assay

For analysis of RhoA activity, 5 cm^2^ of confluent HUVECs at 72 h post-transfection cells were washed with ice-cold PBS and lysed with lysis buffer provided with the G-LISA Activity Assay kit (Cytoskeleton Inc, Denver, CO). After centrifugation of the cell lysates, supernatants were snap-frozen and stored at −80 °C. RhoA activity was determined by using the G-LISA Activity Assay kit, according to manufacturer’s instructions.

### Immunofluorescence imaging

Cells were seeded on 2 cm^2^ and 11 mm coverslips (Menzel, Braunschweig, Germany) coated with 0.5% glutaraldehyde-crosslinked gelatin and transfected as described above. At 72 h post-transfection, culture medium was replaced with prewarmed 4% paraformaldehyde (Merck, Darmstadt, Germany) and incubated on ice for 15 min. Cells were then washed with PBS and permeabilized with 0,5% Triton X-100 (Sigma Aldrich) in PBS and stained with primary antibodies against VE-cadherin overnight at 4°C. After washing cells were incubated with secondary antibodies (Alexa488), rhodamine/phalloidine (direct F-actin staining) and DAPI. Coverslips were mounted with Mowiol4-88/DABCO. Imaging was obtained with a LEICA TCS SP8 confocal microscope system equipped with a 63 × 1.4 NA oil objective (Carl Zeiss, Jena, Germany). Images were analyzed with Leica Application Suit X software and ImageJ (National Institutes of Health).

### DNA constructs

OmickLink-FARP1-Flag (GeneCopoeia EX-E3005-M13-10) was (a kind gift from W.J. Pannekoek, UMC Utrecht and obtained from Labome (Rockville, USA)). Full length FARP1 was amplified by Polymerase Chain Reaction (PCR), using forward primer 5′-GAGATCAGATCTATGGGAGAAATAGAGCAGAGG-3′ and reverse primer 5′-GAGATCGGATCCTTAATACACAAGAGACTCTTTGTGACTC-3′. The PCR product was cut with BglII and BamHI (restriction sites are underlined in the primer sequence) and ligated into the C1-mCherry plasmid, creating C1-mCherry-FARP1 respectively. The FRET biosensors RhoA^30, 81^, Rac1^82^ and Cdc42^31^ were described before.

### HUVEC FRET measurements

HUVECs were transfected as indicated and glass coverslips were placed in Attofluor cell chambers (Thermo Scientific) 16–20 hrs after transfection. FRET images were obtained on a widefield Axiovert 200 M microscope (Carl Zeiss GmbH), equipped with a Plan-Neofluor 40× oil-immersion objective (NA 1.3), a xenon arc lamp with monochromator (Cairn Research, Faversham, Kent, UK) and Metamorph 6.1 software. Images were acquired, using a cooled charged-coupled device camera (Coolsnap HQ, Roper Scientific, Tucson, AZ, USA). CFP was excited at 420 nm (slit width 30 nm) via a 455DCLP dichroic mirror, and emission light was directed to a 470/30 emission filter. Through a rotating emission filter wheel, YFP emission was directed to a 535/30 emission filter. RFP was excited at 570 nm (slit width 10 nm) via a 585CXR dichroic mirror, in turn emission light was redirected to a 620/60 emission filter. FRET image analysis was performed in ImageJ (National Institute of Health), according to previous studies^33, 81, 82^. All image acquisitions were background corrected and bleed-through-corrected (55%) for the CFP leakage into the YFP channel.

### Statistical Analysis

For the siRNA screens, data was read into R^83^ and the data distribution was considered to be acceptable. For each siRNA, its observed values (n = 3) were compared with the non-targeting controls (n = 54) by means of a Student-t test. The produced p-values were corrected for multiple testing by using a Benjamini-Hochberg false-discovery rate^84^. Those siRNAs with FDR < 0.05 were considered significantly different from the non-targeting controls.

For other experiments, data is presented as mean ± SD (standard deviation) unless indicated otherwise. Comparison of two conditions was tested by Student’s t test. P-values <0.05 were considered statistically significant. Statistical analyses were performed using GraphPad Prism Software version 6 (La Jolla, CA).

### Data availability

The datasets generated during and/or analyzed during the current study are available from the corresponding author on reasonable request.

## Electronic supplementary material

Supplementary Information

## References

[1] Mehta D, Malik AB (2006). Signaling mechanisms regulating endothelial permeability. Physiol Rev.

[2] Dudek SM, Garcia JG (2001). Cytoskeletal regulation of pulmonary vascular permeability. J Appl Physiol (1985).

[3] Weis SM (2008). Vascular permeability in cardiovascular disease and cancer. Curr Opin Hematol.

[4] Schnittler HJ, Wilke A, Gress T, Suttorp N, Drenckhahn D (1990). Role of actin and myosin in the control of paracellular permeability in pig, rat and human vascular endothelium. J Physiol.

[5] Risau W, Wolburg H (1990). Development of the blood-brain barrier. Trends in Neurosciences.

[6] Simionescu M, Simionescu N, Palade GE (1975). Segmental differentiations of cell junctions in the vascular endothelium. The microvasculature. J Cell Biol.

[7] Lampugnani MG (2002). VE-cadherin regulates endothelial actin activating Rac and increasing membrane association of Tiam. Molecular biology of the cell.

[8] Moy AB (1996). Histamine and thrombin modulate endothelial focal adhesion through centripetal and centrifugal forces. J Clin Invest.

[9] Moy AB, Blackwell K, Kamath A (2002). Differential effects of histamine and thrombin on endothelial barrier function through actin-myosin tension. Am J Physiol-Heart C.

[10] Vandenbroucke E, Mehta D, Minshall R, Malik AB (2008). Regulation of endothelial junctional permeability. Annals of the New York Academy of Sciences.

[11] Etienne-Manneville S, Hall A (2002). Rho GTPases in cell biology. Nature.

[12] Hall A, Massagué J (2008). Cell regulation. Current opinion in cell biology.

[13] Schmidt A, Hall A (2002). Guanine nucleotide exchange factors for Rho GTPases: turning on the switch. Genes & development.

[14] Hall A (2012). Rho family GTPases. Biochemical Society transactions.

[15] Boulter E (2010). Regulation of Rho GTPase crosstalk, degradation and activity by RhoGDI1. Nature cell biology.

[16] van Buul JD, Geerts D, Huveneers S (2014). Rho GAPs and GEFs: controling switches in endothelial cell adhesion. Cell Adhesion & Migration.

[17] Amado-Azevedo J, Valent ET, Van Nieuw Amerongen GP (2014). Regulation of the endothelial barrier function: a filum granum of cellular forces, Rho-GTPase signaling and microenvironment. Cell and tissue research.

[18] Wójciak-Stothard, B., Potempa, S., Eichholtz, T. & Ridley, A. J. Rho and Rac but not Cdc42 regulate endothelial cell permeability. *Journal of Cell Science* **114** (2001). 10.1242/jcs.114.7.1343 11257000

[19] Van Nieuw Amerongen GP, Van Hinsbergh VWM (2001). Cytoskeletal Effects of Rho-Like Small Guanine Nucleotide-Binding Proteins in the Vascular System. Arteriosclerosis, Thrombosis, and Vascular Biology.

[20] Adamson RH (2002). Rho and rho kinase modulation of barrier properties: cultured endothelial cells and intact microvessels of rats and mice. The Journal of physiology.

[21] Kouklis P, Konstantoulaki M, Vogel S, Broman M, Malik AB (2004). Cdc42 regulates the restoration of endothelial barrier function. Circulation research.

[22] Birukov KG (2009). Small GTPases in mechanosensitive regulation of endothelial barrier. Microvascular research.

[23] Beckers CML, van Hinsbergh VWM, Van Nieuw Amerongen GP (2010). Driving Rho GTPase activity in endothelial cells regulates barrier integrity. Thrombosis and haemostasis.

[24] Gavard J, Patel V, Gutkind JS (2008). Angiopoietin-1 prevents VEGF-induced endothelial permeability by sequestering Src through mDia. Developmental cell.

[25] Vouret-Craviari V, Boquet P, Pouyssegur J, Van Obberghen-Schilling E (1998). Regulation of the actin cytoskeleton by thrombin in human endothelial cells: role of Rho proteins in endothelial barrier function. Mol Biol Cell.

[26] Szulcek R (2013). Localized RhoA GTPase activity regulates dynamics of endothelial monolayer integrity. Cardiovasc Res.

[27] Nobes C, Hall A (1995). Rho, Rac, and Cdc42 GTPases Regulate the Assembly of Multimolecular Focal Complexes Associated with Actin Stress Fibers, Lamellipodia, and Filopodia. Cell.

[28] Rooney C (2010). The Rac activator STEF (Tiam2) regulates cell migration by microtubule-mediated focal adhesion disassembly. EMBO Rep.

[29] Chiu CY (1999). Cloning and characterization of T-cell lymphoma invasion and metastasis 2 (TIAM2), a novel guanine nucleotide exchange factor related to TIAM1. Genomics.

[30] Lin B, Yin T, Wu YI, Inoue T, Levchenko A (2015). Interplay between chemotaxis and contact inhibition of locomotion determines exploratory cell migration. Nature communications.

[31] Kedziora KM (2016). Rapid Remodeling of Invadosomes by G(i)-coupled Receptors: DISSECTING THE ROLE OF Rho GTPases. The Journal of Biological Chemistry.

[32] Heemskerk N (2016). F-actin-rich contractile endothelial pores prevent vascular leakage during leukocyte diapedesis through local RhoA signalling. Nat Commun.

[33] Reinhard NR (2016). Spatiotemporal analysis of RhoA/B/C activation in primary human endothelial cells. Sci Rep.

[34] van Unen J (2016). A New Generation of FRET Sensors for Robust Measurement of Galphai1, Galphai2 and Galphai3 Activation Kinetics in Single Cells. PLoS One.

[35] Matsuo N, Hoshino M, Yoshizawa M, Nabeshima Y (2002). Characterization of STEF, a guanine nucleotide exchange factor for Rac1, required for neurite growth. J Biol Chem.

[36] Lo H-F (2017). Association of dysfunctional synapse defective 1 (SYDE1) with restricted fetal growth – SYDE1 regulates placental cell migration and invasion. The Journal of Pathology.

[37] Feau S, Schoenberger SP, Altman A, Becart S (2013). SLAT regulates CD8+ T cell clonal expansion in a Cdc42- and NFAT1-dependent manner. J Immunol.

[38] Roney KE (2011). Plexin-B2 negatively regulates macrophage motility, Rac, and Cdc42 activation. PLoS One.

[39] Croise P (2016). Cdc42 and Rac1 activity is reduced in human pheochromocytoma and correlates with FARP1 and ARHGEF1 expression. Endocr Relat Cancer.

[40] Wu X, Frost JA (2006). Multiple Rho proteins regulate the subcellular targeting of PAK5. Biochem Biophys Res Commun.

[41] Lim L, Manser E, Leung T, Hall C (1996). Regulation of Phosphorylation Pathways by p21 GTPases. European Journal of Biochemistry.

[42] Zeng Q (2000). Endothelial cell retraction is induced by PAK2 monophosphorylation of myosin II. Journal of cell science.

[43] Wells CM, Jones GE (2010). The emerging importance of group II PAKs. Biochem J.

[44] Matenia D (2005). PAK5 kinase is an inhibitor of MARK/Par-1, which leads to stable microtubules and dynamic actin. Mol Biol Cell.

[45] Cau J, Faure S, Comps M, Delsert C, Morin N (2001). A novel p21-activated kinase binds the actin and microtubule networks and induces microtubule stabilization. The Journal of Cell Biology.

[46] Barry DM (2015). Cdc42 is required for cytoskeletal support of endothelial cell adhesion during blood vessel formation in mice. Development.

[47] Jaiswal M, Dvorsky R, Ahmadian MR (2013). Deciphering the molecular and functional basis of Dbl family proteins: a novel systematic approach toward classification of selective activation of the Rho family proteins. J Biol Chem.

[48] Nir O, Bakal C, Perrimon N, Berger B (2010). Inference of RhoGAP/GTPase regulation using single-cell morphological data from a combinatorial RNAi screen. Genome research.

[49] Pascual-Vargas P (2017). RNAi screens for Rho GTPase regulators of cell shape and YAP/TAZ localisation in triple negative breast cancer. Sci Data.

[50] Wallace SW, Magalhaes A, Hall A (2011). The Rho target PRK2 regulates apical junction formation in human bronchial epithelial cells. Molecular and cellular biology.

[51] Abiko H (2015). Rho guanine nucleotide exchange factors involved in cyclic-stretch-induced reorientation of vascular endothelial cells. J Cell Sci.

[52] Sanz-Moreno V (2008). Rac activation and inactivation control plasticity of tumor cell movement. Cell.

[53] Williams SP (2017). Systematic high-content genome-wide RNAi screens of endothelial cell migration and morphology. Sci Data.

[54] Vaqué JP (2013). A genome-wide RNAi screen reveals a Trio-regulated Rho GTPase circuitry transducing GPCR-initiated mitogenic signals. Molecular cell.

[55] Marcos-Ramiro B (2016). RhoB controls endothelial barrier recovery by inhibiting Rac1 trafficking to the cell border. The Journal of Cell Biology.

[56] Van Nieuw Amerongen GP, Draijer R, Vermeer MA, van Hinsbergh VWM (1998). Transient and Prolonged Increase in Endothelial Permeability Induced by Histamine and Thrombin: Role of Protein Kinases, Calcium, and RhoA. Circulation Research.

[57] Birukova AA (2006). GEF-H1 is involved in agonist-induced human pulmonary endothelial barrier dysfunction. Am J Physiol Lung Cell Mol Physiol.

[58] Mikelis CM (2013). PDZ-RhoGEF and LARG are essential for embryonic development and provide a link between thrombin and LPA receptors and Rho activation. The Journal of biological chemistry.

[59] Gavard J, Gutkind JS (2006). VEGF controls endothelial-cell permeability by promoting the beta-arrestin-dependent endocytosis of VE-cadherin. Nature cell biology.

[60] Blangy A (2000). TrioGEF1 controls Rac- and Cdc42-dependent cell structures through the direct activation of RhoG. Journal of Cell Science.

[61] Prieto-Sanchez RM, Bustelo XR (2003). Structural basis for the signaling specificity of RhoG and Rac1 GTPases. J Biol Chem.

[62] Knezevic II (2009). Tiam1 and Rac1 are required for platelet-activating factor-induced endothelial junctional disassembly and increase in vascular permeability. The Journal of biological chemistry.

[63] Zhao ZY (2013). TIAM2 enhances non-small cell lung cancer cell invasion and motility. Asian Pac J Cancer Prev.

[64] Keese CR, Wegener J, Walker SR, Giaever I (2004). Electrical wound-healing assay for cells *in vitro*. Proceedings of the National Academy of Sciences of the United States of America.

[65] Schiller HB, Fässler R (2013). Mechanosensitivity and compositional dynamics of cell-matrix adhesions. EMBO reports.

[66] Szulcek R, Bogaard HJ, van Nieuw Amerongen GP (2014). Electric cell-substrate impedance sensing for the quantification of endothelial proliferation, barrier function, and motility. J Vis Exp.

[67] Skaria T, Burgener J, Bachli E, Schoedon G (2016). IL-4 Causes Hyperpermeability of Vascular Endothelial Cells through Wnt5A Signaling. PLoS One.

[68] Wójciak-Stothard B, Entwistle A, Garg R, Ridley AJ (1998). Regulation of TNF-α-induced reorganization of the actin cytoskeleton and cell-cell junctions by Rho, Rac, and Cdc42 in human endothelial cells. Journal of Cellular Physiology.

[69] Michiels F, Habets GGM, Stam JC, van der Kammen RA, Collard JG (1995). A role for Rac in Tiaml-induced membrane ruffling and invasion. Nature.

[70] Schuebel KE, Movilla N, Rosa JL, Bustelo XR (1998). Phosphorylation-dependent and constitutive activation of Rho proteins by wild-type and oncogenic Vav-2. EMBO J.

[71] Movilla N, Bustelo XR (1999). Biological and regulatory properties of Vav-3, a new member of the Vav family of oncoproteins. Mol Cell Biol.

[72] de Kreuk BJ (2013). The human minor histocompatibility antigen 1 is a RhoGAP. PLoS One.

[73] Huang RY (2015). Functional relevance of a six mesenchymal gene signature in epithelial-mesenchymal transition (EMT) reversal by the triple angiokinase inhibitor, nintedanib (BIBF1120). Oncotarget.

[74] Hernandez-Garcia R, Iruela-Arispe ML, Reyes-Cruz G, Vazquez-Prado J (2015). Endothelial RhoGEFs: A systematic analysis of their expression profiles in VEGF-stimulated and tumor endothelial cells. Vascul Pharmacol.

[75] Cheadle L, Biederer T (2012). The novel synaptogenic protein Farp1 links postsynaptic cytoskeletal dynamics and transsynaptic organization. The Journal of cell biology.

[76] Koyano Y (2001). Chondrocyte-derived ezrin-like domain containing protein (CDEP), a rho guanine nucleotide exchange factor, is inducible in chondrocytes by parathyroid hormone and cyclic AMP and has transforming activity in NIH3T3 Cells. Osteoarthritis and Cartilage.

[77] He X, Kuo Y-C, Rosche TJ, Zhang X (2013). Structural basis for auto-inhibition of theP guanine nucleotide exchange factor FARP2. Structure (London, England: 1993).

[78] V N Amerongen GP, Koolwijk P, Versteilen AMG, van Hinsbergh VWM (2003). Involvement of RhoA/Rho Kinase Signaling in VEGF-Induced Endothelial Cell Migration and Angiogenesis *In Vitro*. Arteriosclerosis, Thrombosis, and Vascular Biology.

[79] Hannus M (2014). siPools: highly complex but accurately defined siRNA pools eliminate off-target effects. Nucleic Acids Res.

[80] Parsons BD, Schindler A, Evans DH, Foley E (2009). A direct phenotypic comparison of siRNA pools and multiple individual duplexes in a functional assay. PLoS One.

[81] van Unen J (2015). Plasma membrane restricted RhoGEF activity is sufficient for RhoA-mediated actin polymerization. Scientific Reports.

[82] Timmerman I (2015). A local VE-cadherin and Trio-based signaling complex stabilizes endothelial junctions through Rac1. Journal of Cell Science.

[83] R: A Language and Environment for Statistical Computing (R Foundation for Statistical Computing, Vienna, Austria, 2008).

[84] Benjamini Y, Hochberg Y (1995). Controlling the False Discovery Rate: A Practical and Powerful Approach to Multiple Testing. Journal of the Royal Statistical Society.

