# Supplementary material for: A CDC42-centered signaling unit is a dominant positive regulator of endothelial integrity
Source: Sci Rep. 2017 Aug 31;7:10132. doi: 10.1038/s41598-017-10392-0 (PMC5579287; doi:10.1038/s41598-017-10392-0)

Supplementary figures to the manuscript

Title: A CDC42-centered signaling unit is a dominant positive regulator of endothelial integrity

Authors: Joana Amado-Azevedo[1], N. R. Reinhard[2], J. van Bezu[1], R. X. de Menezes[3], V. W. van Beusechem[4], G. P. van Nieuw Amerongen[1], V. W. M. van Hinsbergh[1], P. L. Hordijk[1*]

Author affiliations:
[1]Department of Physiology, Institute of Cardiovascular Research, VU University Medical Center, Amsterdam, The Netherlands
[2]Department of Molecular Cytology, Swammerdam Institute for Life Sciences, van Leeuwenhoek Centre for Advanced Microscopy, University of Amsterdam, Amsterdam, The Netherlands
[3]Department of Epidemiology and Biostatistics, VU University Medical Center, Amsterdam, The Netherlands
[4]Department of Medical Oncology, RNA Interference Functional Oncogenomics Laboratory, VU University Medical Center, Amsterdam, The Netherlands

*corresponding author: p.hordijk@vumc.nl

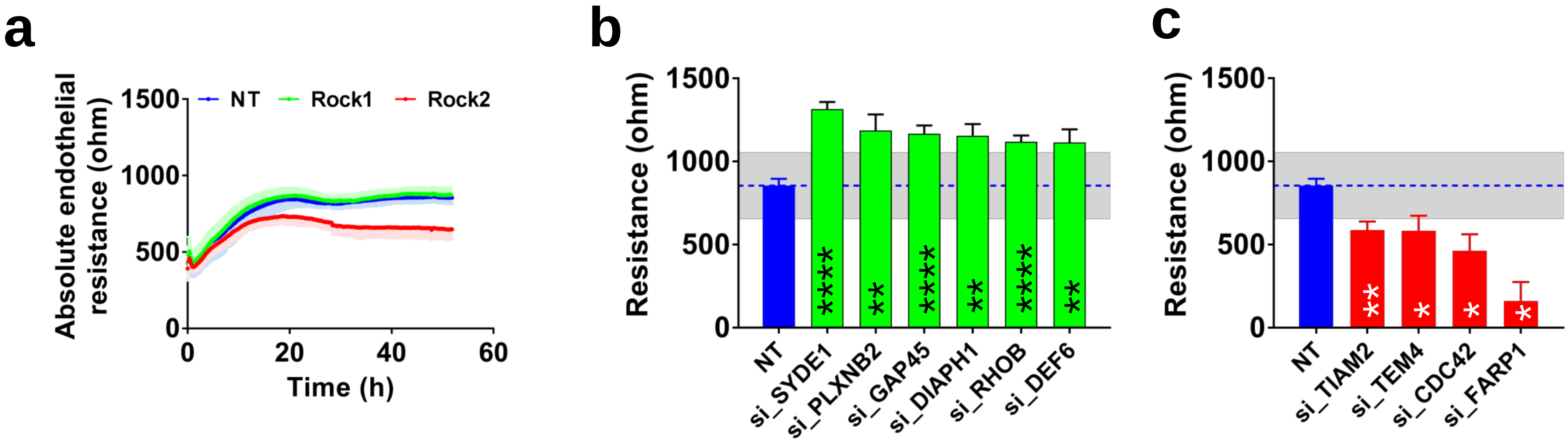

Supplementary Figure 1.
(**a**) Endothelial barrier resistance curves of siRNA controls, 16h post-transfection used in the three screens. In red, the ROCK2 siRNA used as positive control, in blue the NT siRNA control and in green, ROCK1 siRNA used as negative control (**b**) Basal endothelial barrier resistance levels at 72h post-transfection of the six genes that, upon knock-down, significantly enhanced the endothelial barrier (n=3) when compared to NT siRNA controls (n=18).***P<0,001, **P<0,01, *P<0.05 in two-tailed Student's t-test. Error bars depict mean ± SEM. (**c**) Basal endothelial barrier resistance levels at 72h post-transfection of the four genes that upon knock-down, significantly decreased endothelial barrier resistance (n=3) when compared to NT siRNA controls (n=18). **P<0,01 and *P<0.05 in two-tailed Student's t-test. Error bars depict mean ± SEM.

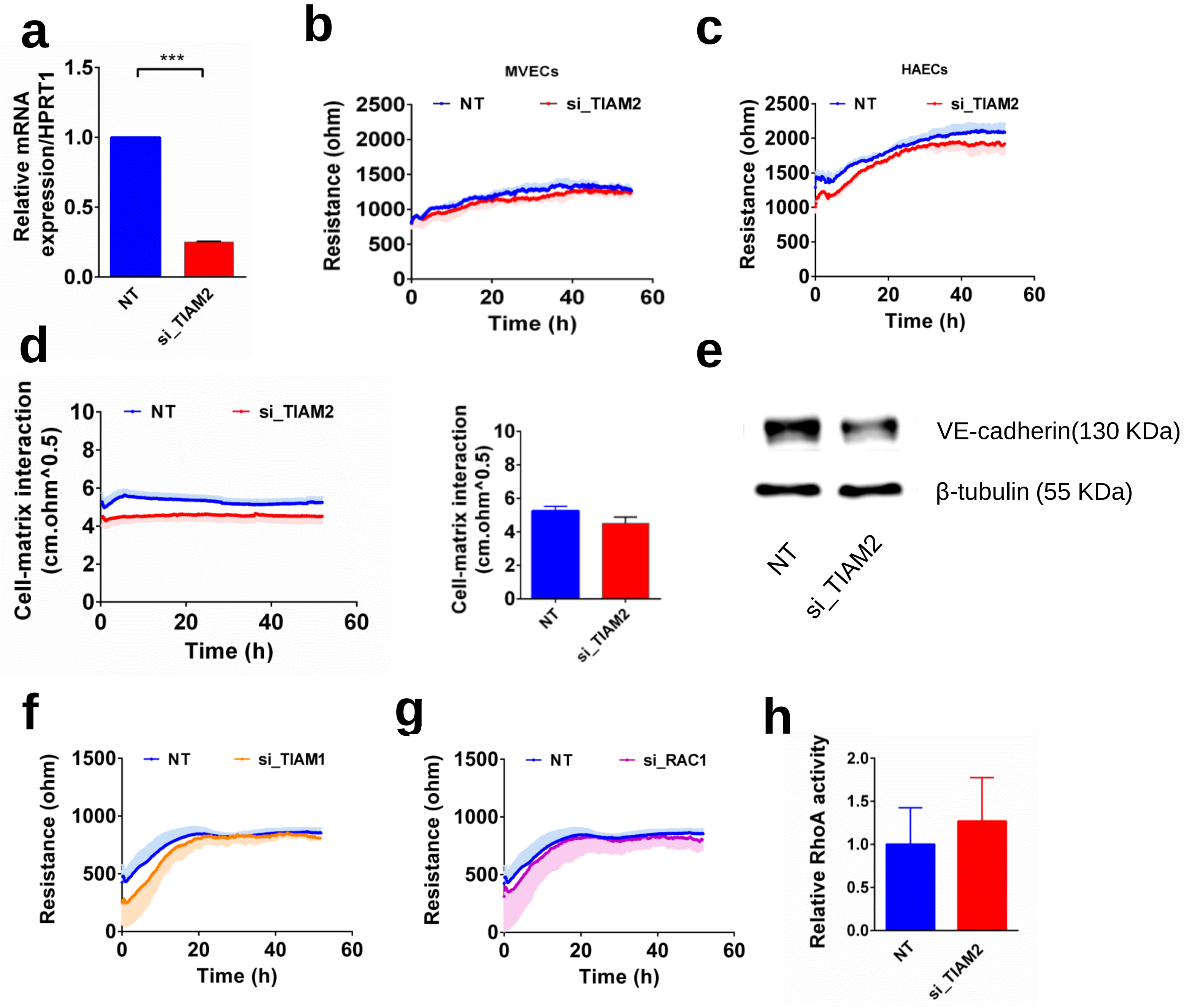

Supplementary Figure 2.
(**a**) Transfection efficiency of si_TIAM2 as determined by qPCR. ***P<0.001 in Student T-test (n=3); (**b**) Absolute endothelial resistance of MVECs transfected with si_TIAM2 and non-targeting siRNA control (n=2); (**c**) Absolute endothelial resistance of HAECs transfected with si_TIAM2 and non-targeting siRNA control (n=2); (**d**) Absolute endothelial electrical resistance attributable to cell-matrix interaction of monolayers of HUVECs transfected with si_TIAM2 and NT siRNA control 16h after transfection. Bar graph depicts quantification at time-point 72h post-transfection. Not significant in two-tailed Student's t-test (n=3 ± SEM); (**e**) Western blot analysis of the junctional proteins VE-cadherin of HUVECs transfected with si_TIAM2 and NT siRNA control (n=3) (full length blot are presented in the supplementary information section); (**f**) Absolute endothelial resistance of HUVECs transfected with si_TIAM1 and NT siRNA control, 16h post-transfection (n=3); (**g**) Absolute endothelial resistance of HUVECs transfected with si_Rac1 and NT siRNA control, 16h post-transfection (n=3); (**h**) Relative RhoA activity levels of HUVECs transfected with si_TIAM2 and non-targeting control siRNA at baseline. Not significant in two-tailed Student's t-test (n=3).

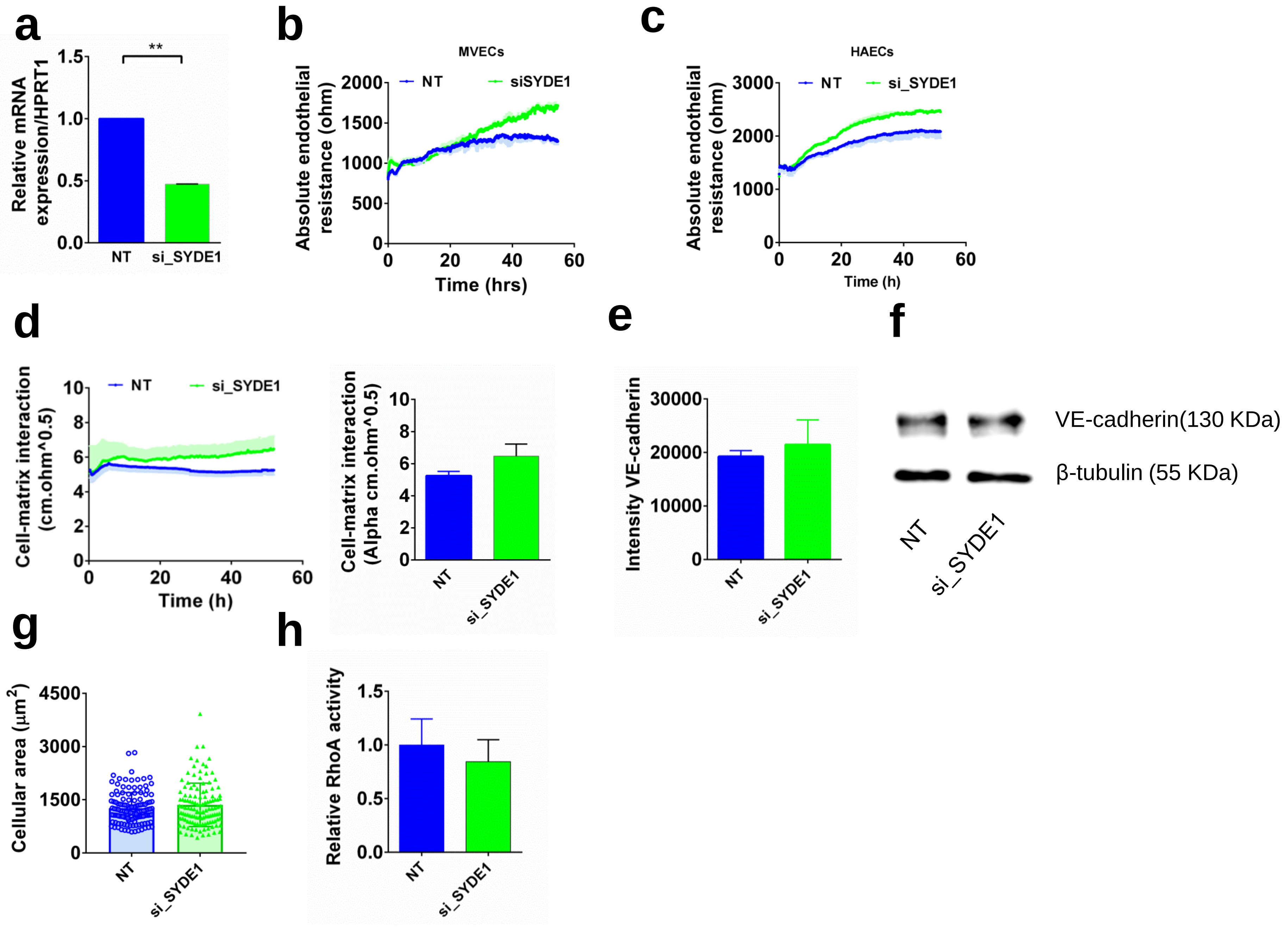

Supplementary Figure 3.

(**a**) Transfection efficiency of si_SYDE1 determined by qPCR. **P<0.01 in Student T-test (n=3); (**b**) Absolute endothelial resistance of MVECs transfected with si_SYDE1 and non-targeting siRNA control (n=2); (**c**) Absolute endothelial resistance of HAECs transfected with si_SYDE1 and non-targeting siRNA control (n=2); (**d**) Absolute endothelial electrical resistance attributable to cell-matrix interaction (Alpha) of monolayers of HUVECs transfected with si_SYDE1 and NT siRNA control 16h after transfection. Respective quantification of last time-point. Not significant in two-tailed Student's t-test (n=3 ± SEM); (**e**) Quantification of total VE-cadherin fluorescence of immunostaining slides of HUVECs transfected with si_SYDE1 and NT siRNA control. Not significant in two-tailed Student's t-test (n=3); (**f**) Western blot analysis of the junctional protein VE-cadherin of HUVECs transfected with si_SYDE1 and NT siRNA control, representative image (n=3) (full length blot are presented in the supplementary information section); (**g**) Quantification of cellular area of HUVECs transfected with si_SYDE1 (n=124) and NT siRNA control (n=132) at 72h post-transfection (Not significant in two-tailed Student's t-test (n=3); (**h**) Relative RhoA activity levels of HUVECs transfected with si_SYDE1 and non-targeting control siRNA at baseline. Not significant in two-tailed Student's t-test (n=3).

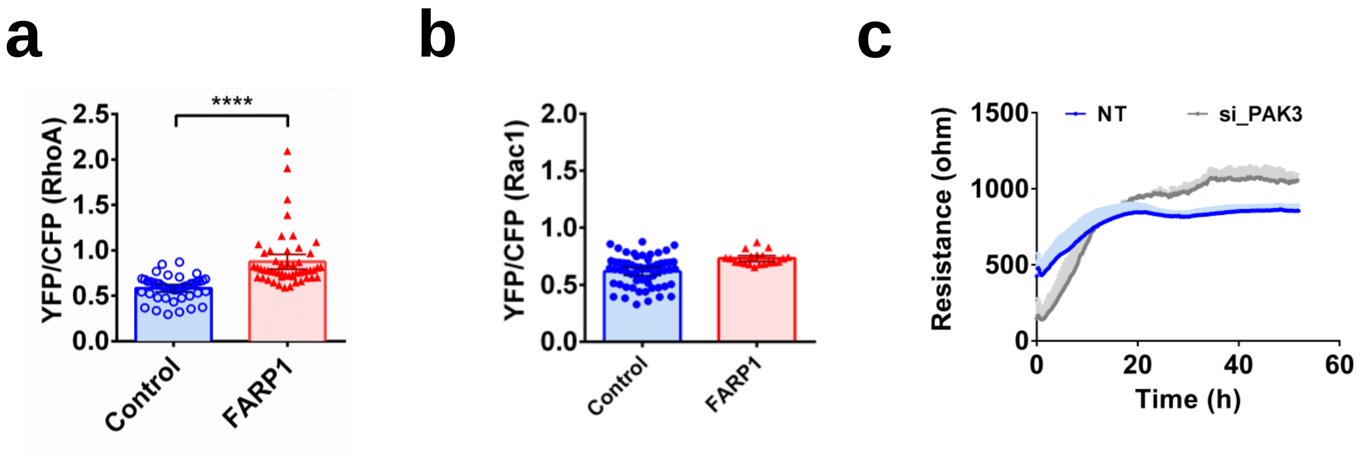

Supplementary Figure 4.
(**a**) YFP/CFP ratios of RhoA FRET sensor-expressing HUVECs co-expressing either mCherry control (n=44) or mCherry FARP1 (n=52). Error bars depict 95%CI. ****P<0.0001 in two-tailed Student's t-test; (**b**) YFP/CFP ratios of Rac1 FRET sensor-expressing HUVECs co-expressing either mCherry control (n=59) or mCherry FARP1 (n=20). Error bars depict 95%CI; (**c**) Absolute endothelial resistance of HUVECs transfected with si_Pak3 and NT siRNA control, 16h post-transfection (n=3);

Supplementary Table S1. – siRNA library overview.

	Gene Symbol	Gene ID	Accession # (NM_)
1	**AKAP13**	11214	NM_006738
2	**ALS2**	57679	NM_020919
3	**ARHGEF1**	9138	NM_004706
4	**ARHGEF10**	9639	NM_014629
5	**ARHGEF10L**	55160	NM_018125
6	**ARHGEF11**	9826	NM_198236
7	**ARHGEF12**	23365	NM_015313
8	**ARHGEF15**	22899	NM_173728
9	**ARHGEF16**	27237	NM_014448
10	**ARHGEF17**	9828	NM_014786
11	**ARHGEF18**	23370	NM_015318
12	**ARHGEF19**	128272	NM_153213
13	**ARHGEF2**	9181	NM_004723
14	**ARHGEF25**	115557	NM_182947
15	**ARHGEF26**	26084	NM_015595
16	**ARHGEF28**	64283	NM_001080479
17	**ARHGEF3**	50650	NM_019555
18	**ARHGEF37**	389337	NM_001001669
19	**ARHGEF38**	54848	NM_017700
20	**ARHGEF39**	84904	NM_032818
21	**ARHGEF4**	50649	NM_015320
22	**ARHGEF40**	55701	NM_018071
23	**ARHGEF5**	7984	NM_005435
24	**ARHGEF6**	9459	NM_004840
25	**ARHGEF7**	8874	NM_003899
26	**ARHGEF9**	23229	NM_015185
27	**CD44**	960	NM_000610
28	**CTNND2**	1501	NM_001332
29	**CYSLTR2**	57105	NM_020377
30	**DEF6**	50619	NM_022047
31	**DNMBP**	23268	NM_015221
32	**DOCK1**	1793	NM_001380
33	**DOCK10**	55619	NM_014689
34	**DOCK11**	139818	NM_144658
35	**DOCK2**	1794	NM_004946
36	**DOCK3**	1795	NM_004947
37	**DOCK4**	9732	NM_014705
38	**DOCK5**	80005	NM_024940
39	**DOCK6**	57572	NM_020812
40	**DOCK7**	85440	NM_033407
41	**DOCK8**	81704	NM_203447
42	**DOCK9**	23348	NM_015296
43	**ECT2**	1894	NM_018098
44	**EGFR**	1956	NM_005228
45	**FARP1**	10160	NM_005766
46	**FARP2**	9855	NM_014808
47	**FGD1**	2245	NM_004463
48	**FGD2**	221472	NM_173558
49	**FGD3**	89846	NM_033086
50	**FGD4**	121512	NM_139241
51	**FGD5**	152273	NM_152536
52	**FGD6**	55785	NM_018351
53	**GNA12**	2768	NM_007353
54	**GNA13**	10672	NM_006572
55	**GNAQ**	2776	NM_002072
56	**GNB1**	2782	NM_002074
57	**GNG2**	54331	NM_053064
58	**IGDCC4**	57722	NM_020962
59	**ITSN1**	6453	NM_003024
60	**ITSN2**	50618	NM_006277
61	**LMOD1**	25802	NM_012134
62	**MCF2**	4168	NM_005369
63	**MCF2L**	23263	NM_024979
64	**MCF2L2**	23101	NM_015078
65	**NET1**	10276	NM_001047160
66	**NGEF**	25791	NM_019850
67	**OBSCN**	84033	NM_052843
68	**OBSL1**	23363	NM_015311
69	**PAK1IP1**	55003	NM_017906
70	**PLEK**	5341	NM_002664
71	**PLEKHF1**	79156	NM_024310
72	**PLEKHF2**	79666	NM_024613
73	**PLEKHG1**	57480	NM_001029884
74	**PLEKHG2**	64857	NM_022835
75	**PLEKHG3**	26030	NM_015549
76	**PLEKHG4**	25894	NM_015432
77	**PLEKHG4B**	153478	NM_052909
78	**PLEKHG5**	57449	NM_020631
79	**PLEKHG6**	55200	NM_018173
80	**PLEKHG7**	440107	NM_001004330
81	**PLXNB1**	5364	NM_002673
82	**PLXNB2**	23654	NM_012401
83	**PLXNB3**	5365	NM_005393
84	**PREX1**	57580	NM_020820
85	**PREX2**	80243	NM_025170
86	**RAPGEF2**	9693	NM_014247
87	**RASGRF1**	5923	NM_002891
88	**RASGRF2**	5924	NM_006909
89	**SOS1**	6654	NM_005633
90	**SOS2**	6655	NM_006939
91	**SPATA13**	221178	NM_153023

92	**SWAP70**	23075	NM_015055
93	**TIAM1**	7074	NM_003253
94	**TIAM2**	26230	NM_012454
95	**TRIO**	7204	NM_007118
96	**VAV1**	7409	NM_005428
97	**VAV2**	7410	NM_003371
98	**VAV3**	10451	NM_006113
99	**ARHGDIA**	396	NM_004309
100	**ARHGDIB**	397	NM_001175
101	**ARHGDIG**	398	NM_001176
102	**ARPC1A**	10552	NM_006409
103	**ARPC1B**	10095	NM_005720
104	**ARPC2**	10109	NM_005731
105	**BAIAP2**	10458	NM_006340
106	**CDC42EP1**	11135	NM_152243
107	**CDC42EP2**	10435	NM_006779
108	**CDC42EP3**	10602	NM_006449
109	**CDC42EP4**	23580	NM_012121
110	**CDC42EP5**	148170	NM_145057
111	**CFL1**	1072	NM_005507
112	**CFL2**	1073	NM_138638
113	**Cit**	11113	NM_007174
114	**Cnksr1**	10256	NM_006314
115	**CNN1**	1264	NM_001299
116	**DgkQ**	1609	NM_001347
117	**Diaph1**	1729	NM_005219
118	**Diaph2**	1730	NM_006729
119	**Diaph3**	81624	NM_001042517
120	**EEF1A1**	1915	NM_001402
121	**ELMO1**	9844	NM_014800
122	**ELMO2**	63916	NM_133171
123	**ELMO3**	79767	NM_024712
124	**EZR**	7430	NM_003379
125	**FlnA**	2316	NM_001456
126	**GDI1**	2664	NM_001493
127	**GDI2**	2665	NM_001494
128	**GSN**	2934	NM_198252
129	**Itpr1**	3708	NM_001099952
130	**KcnA2**	3737	NM_004974
131	**Ktn1**	3895	NM_001079521
132	**LIMK1**	3984	NM_002314
133	**MARCKS**	4082	NM_002356
134	**MLC1**	23209	NM_015166
135	**MSN**	4478	NM_002444
136	**MYBPH**	4608	NM_004997
137	**PAK1**	5058	NM_002576

138	**PAK2**	5062	NM_002577
139	**PAK3**	5063	NM_001128166
140	**PAK4**	10298	NM_005884
141	**PAK6**	56924	NM_020168
142	**PAK7**	57144	NM_020341
143	**PFN1**	5216	NM_005022
144	**PFN2**	5217	NM_002628
145	**PIKFYVE**	200576	NM_015040
146	**Pkn1**	5585	NM_002741
147	**Pkn2**	5586	NM_006256
148	**PlcG1**	5335	NM_002660
149	**Pld1**	5337	NM_002662
150	**PLK1**	5347	NM_005030
151	**PLK2**	10769	NM_006622
152	**PLK3**	1263	NM_004073
153	**PLK4**	10733	NM_014264
154	**PLK5**	126520	NM_001243079
155	**Ppp1r12A**	4659	NM_002480
156	**PPP1R14A**	94274	NM_033256
157	**PrkcA**	5578	NM_002737
158	**PTK2**	5747	NM_005607
159	**RDX**	5962	NM_001260492
160	**RHO**	6010	NM_000539
161	**Rhpn1**	114822	NM_052924
162	**Rhpn2**	85415	NM_033103
163	**Rock1**	6093	NM_005406
164	**Rock2**	9475	NM_004850
165	**RTKN**	6242	NM_033046
166	**RTKN2**	219790	NM_145307
167	**SEPT1**	1731	NM_052838
168	**SEPT2**	4735	NM_006155
169	**SEPT3**	55964	NM_019106
170	**SLC9A1**	6548	NM_003047
171	**SLC9A3R1**	9368	NM_004252
172	**TP63**	8626	NM_003722
173	**TRIP10**	9322	NM_004240
174	**VCL**	7414	NM_014000
175	**VIL1**	7429	NM_007127
176	**VILL**	50853	NM_015873
177	**WAS**	7454	NM_000377
178	**WASF1**	8936	NM_003931
179	**ABR**	29	NM_001092
180	**ARAP1**	116985	NM_015242
181	**ARAP2**	116984	NM_015230
182	**ARAP3**	64411	NM_022481
183	**ARHGAP1**	392	NM_004308

184	**ARHGAP10**	79658	NM_024605
185	**ARHGAP11A**	9824	NM_014783
186	**ARHGAP11B**	89839	NM_001039841
187	**ARHGAP12**	94134	NM_018287
188	**ARHGAP15**	55843	NM_018460
189	**ARHGAP17**	55114	NM_018054
190	**ARHGAP18**	93663	NM_033515
191	**ARHGAP19**	84986	NM_032900
192	**ARHGAP20**	57569	NM_020809
193	**ARHGAP21**	57584	NM_020824
194	**ARHGAP22**	58504	NM_021226
195	**ARHGAP23**	57636	NM_001199417
196	**ARHGAP24**	83478	NM_031305
197	**ARHGAP25**	9938	NM_014882
198	**ARHGAP26**	23092	NM_015071
199	**ARHGAP27**	201176	NM_199282
200	**ARHGAP28**	79822	NM_001010000
201	**ARHGAP29**	9411	NM_004815
202	**ARHGAP30**	257106	NM_181720
203	**ARHGAP31**	57514	NM_020754
204	**ARHGAP32**	9743	NM_014715
205	**ARHGAP33**	115703	NM_052948
206	**ARHGAP35**	2909	NM_004491
207	**ARHGAP36**	158763	NM_144967
208	**ARHGAP39**	80728	NM_025251
209	**ARHGAP4**	393	NM_001666
210	**ARHGAP40**	343578	NM_001164431
211	**ARHGAP42**	143872	NM_152432
212	**ARHGAP44**	9912	NM_014859
213	**ARHGAP5**	394	NM_001173
214	**ARHGAP6**	395	NM_013427
215	**ARHGAP8**	23779	NM_001017526
216	**ARHGAP9**	64333	NM_032496
217	**BCR**	613	NM_004327
218	**BNIP2**	663	NM_004330
219	**CHN1**	1123	NM_001822
220	**CHN2**	1124	NM_004067
221	**DEPDC1**	55635	NM_017779
222	**DEPDC1B**	55789	NM_018369
223	**FAM13A**	10144	NM_014883
224	**FAM13B**	51306	NM_016603
225	**GMIP**	51291	NM_016573
226	**HMHA1**	23526	NM_012292
227	**INPP5B**	3633	NM_005540
228	**IQGAP1**	8826	NM_003870
229	**IQGAP2**	10788	NM_006633
230	**MYO3A**	53904	NM_017433
231	**MYO3B**	140469	NM_138995
232	**MYO9A**	4649	NM_006901
233	**MYO9B**	4650	NM_004145
234	**OCRL**	4952	NM_000276
235	**OPHN1**	4983	NM_002547
236	**PIK3R1**	5295	NM_181504
237	**PIK3R2**	5296	NM_005027
238	**RACGAP1**	29127	NM_013277
239	**RALBP1**	10928	NM_006788
240	**SH3BP1**	23616	NM_018957
241	**SRGAP1**	57522	NM_020762
242	**SRGAP2**	23380	NM_015326
243	**SRGAP3**	9901	NM_014850
244	**STARD13**	90627	NM_052851
245	**STARD8**	9754	NM_014725
246	**SYDE1**	85360	NM_033025
247	**SYDE2**	84144	NM_032184
248	**TAGAP**	117289	NM_054114
249	**CDC42**	998	NM_001791
250	**RAC1**	5879	NM_006908
251	**RAC2**	5880	NM_002872
252	**RAC3**	5881	NM_005052
253	**RhoA**	387	NM_001664
254	**RHOB**	388	NM_004040
255	**RHOBTB1**	9886	NM_014836
256	**RHOBTB2**	23221	NM_001160036
257	**RHOBTB3**	22836	NM_014899
258	**RHOC**	389	NM_175744
259	**RHOD**	29984	NM_014578
260	**RHOF**	54509	NM_019034
261	**RHOG**	391	NM_001665
262	**RHOJ**	57381	NM_020663
263	**RHOQ**	23433	NM_012249
264	**RHOT1**	55288	NM_018307
265	**RHOT2**	89941	NM_138769
266	**RHOU**	58480	NM_021205
267	**RHOV**	171177	NM_133639
268	**RND1**	27289	NM_014470
269	**RND2**	8153	NM_005440
270	**RND3**	390	NM_005168

RhoGEFs
RhoGDIs
Effectors
RhoGAPs
RhoGTPases

Full length blot for VE-cadherin and Tubulin shown on supplemental figure 2e

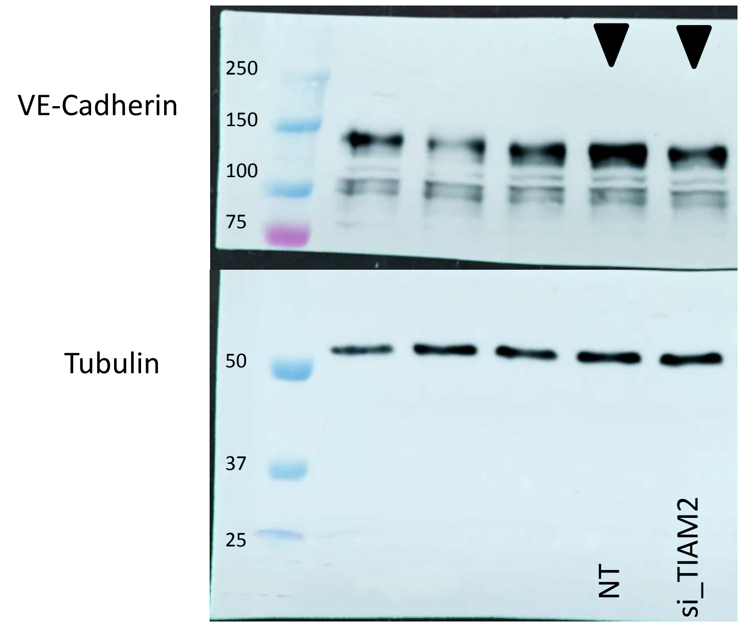

Full length blot for VE-cadherin and Tubulin shown on supplemental figure 3f

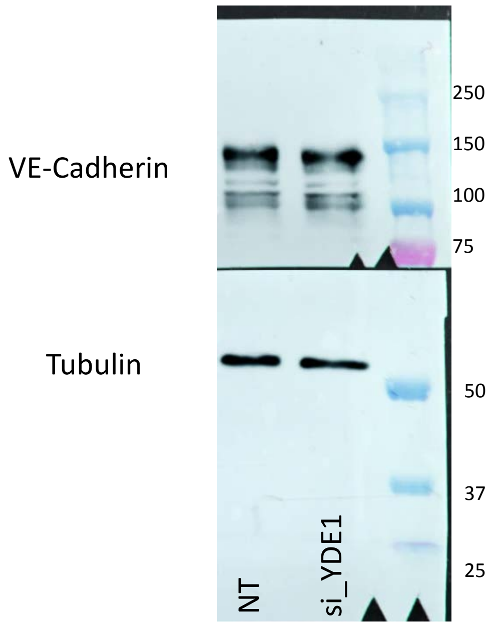

Supplement: Supplementary file 1 — Supplementary Information [file 41598_2017_10392_MOESM1_ESM.pdf]
